# Intraoperative methadone for postoperative pain in adult patients undergoing tonsillectomy—a randomised controlled trial

**DOI:** 10.1016/j.bjao.2025.100418

**Published:** 2025-05-26

**Authors:** Michael Bøndergaard, Peter Gaarsdal Uhrbrand, Tutku Karaca, Marianne Rhode, Thomas Kjærgaard, Rene Thunberg Svendsen, Tejs Ehlers Klug, Lone Nikolajsen, Kristian Dahl Friesgaard

**Affiliations:** 1 Department of Anaesthesiology and Intensive Care, Randers Regional Hospital, Randers, Denmark; 2 Department of Anaesthesiology and Intensive Care, Aarhus University Hospital, Aarhus, Denmark; 3 Department of Otorhinolaryngology, Head & Neck Surgery, Aarhus University Hospital, Aarhus, Denmark; 4 Department of Clinical Medicine, Aarhus University, Denmark; 5 Department of Anaesthesiology and Intensive Care, Horsens Regional Hospital, Denmark

**Keywords:** acute postoperative pain, methadone, pain management, postoperative, tonsillectomy

## Abstract

**Background:**

Tonsillectomy is a common procedure often associated with severe postoperative pain. This study hypothesised that methadone would provide superior postoperative pain relief and reduced opioid consumption compared with fentanyl.

**Methods:**

A total of 120 adult patients undergoing elective bilateral tonsillectomy were randomly allocated to receive either methadone (0.2 mg kg^−1^; *n*=62) or fentanyl (3 μg kg^−1^; *n*=58) after anaesthesia induction. Joint primary outcomes were pain intensity (numeric rating scale, 0–10) at swallowing upon postanaesthesia care unit (PACU) arrival and cumulative opioid consumption (oral morphine equivalents) over 5 postoperative days. Secondary outcomes included pain at swallowing, PACU and hospital stay duration, sedation at 4 h, patient satisfaction at days 1 and 7, postoperative nausea/vomiting (PONV) on days 1–3, and PACU adverse events.

**Results:**

Cumulative 5-day opioid consumption was lower in the methadone group (30 mg, inter-quartile range [IQR] 10–50 mg) *vs* the fentanyl group (49 mg, IQR 29–80 mg, *P*=0.002), driven by reduced use on day 1. Pain intensity was lower in the methadone group compared with the fentanyl group in the PACU (4, IQR 3–5 *vs* 5, IQR 4–7, *P*=0.0004), at 24 h (*P*=0.005) and 48 h (*P*=0.03). More patients in the methadone group experienced moderate to severe PONV at 24 h (45.0%, *vs* 14.1%, *P*=0.001), 48 h (43.3% *vs* 17.6%, *P*=0.005), and 72 h (33.9% *vs* 18.2%, *P*=0.03). Secondary outcomes, including patient satisfaction, sedation, and discharge times, did not differ significantly.

**Conclusion:**

Methadone reduced opioid consumption and pain intensity but increased PONV. Identifying risk factors for both severe postoperative pain and PONV may help guide patient selection for methadone use.

**Clinical trial registration:**

NCT05445856, EudraCT ID 2022-002496-11.

Tonsillectomy is one of the most commonly performed operations in Europe and a substantial number of adult patients experience considerable postoperative pain.^1^ With advances in surgical techniques and anaesthesia, tonsillectomy, like many other procedures, is increasingly being performed as day surgery. Perioperative anaesthetic innovations, including short-acting total intravenous anaesthesia and multimodal analgesia, help reduce opioid administration and minimise postoperative side-effects.^2^ However, intense surgical stimuli and anatomical regions inaccessible to multimodal techniques, such as regional anaesthesia, highlight that some surgical populations still require opioids to relieve postoperative pain. Rather than administering multiple, and often inadequate doses of short-acting opioids after operation, a single intraoperative dose of a long-acting opioid could potentially cover the first 24 h after surgery, during which pain is known to be the most severe.^1^ This approach could, in theory, reduce the total opioid consumption and potentially minimise unwanted side-effects.

Methadone is an opioid with several pharmacological advantages, including long elimination half-life. It has been shown to inhibit serotonin and norepinephrine reuptake^3^ and to interact with *N*-methyl-d-aspartate (NMDA) receptors *in vitro*,^4^ although the clinical relevance of these mechanisms remains uncertain. These properties may contribute to its use in treating chronic and neuropathic pain.^5^, ^6^, ^7^ Recently, methadone has gained attention in the perioperative setting, where reduced pain and lower postoperative opioid consumption are desired outcomes.^8^ However, the number of studies on methadone in this context remains limited, with most focusing on major surgery such as open cardiac surgery^9^ and spinal fusion.^10^ The use of methadone in adult tonsillectomy patients has not been well studied, despite the need for better pain control in this population.^1^ The aim of this randomised controlled trial was to investigate the effect of a single i.v. dose of methadone on postoperative pain, opioid consumption, and side-effects, compared with fentanyl in adult patients scheduled for tonsillectomy. We hypothesised that methadone would reduce pain and postoperative opioid requirements compared with fentanyl, with no difference in side-effects between the two groups.

## Methods

### Study design and study population

This investigator-initiated, prospective, randomised controlled trial was reported in alignment with the Consolidated Standards of Reporting Trials (CONSORT) reporting guidelines.^11^ The trial was carried out in agreement with the Helsinki II Declaration and guidelines for Good Clinical Practice (GCP) and supervised by the GCP unit at Aarhus University Hospital, Denmark. Approval was obtained by relevant authorities, including the Danish Data Protection Agency (ID 1-16-02-254-22), The Danish Research Ethics Committees (ID 2209451), and the Danish Health and Medicines Authority (ID 2022-002496-11). The trial was registered before patient enrolment at clinicaltrials.gov (NCT05445856, principal investigator: Michael Bøndergaard, Date of registration: June 30 2022) and the European Clinical Trials Database (EudraCT ID 2022-002496-11). Written informed consent was obtained from all subjects participating in the trial.

Adult patients (age >18 years) scheduled for elective bilateral tonsillectomy at Randers Regional Hospital, Denmark, were approached for study participation with the following exclusion criteria applied: American Society of Anesthesiologists (ASA) physical status 4 or 5,^12^ allergy to study drugs, daily opioids use at least 7 days before surgery, inability to provide informed consent, known severe respiratory insufficiency, heart failure, acute alcohol intoxication/delirium tremens, increased intracranial pressure, acute liver disease or insufficiency, kidney insufficiency, pregnancy, breastfeeding, and treatment with rifampicin or any drug prolonging the QT interval on electrocardiogram.

### Randomisation and blinding

Participants were randomly allocated in a 1:1 ratio in varying block sizes (between 4 and 8) to receive either intraoperative methadone (Streuli Pharma AG, Uznach, Switzerland) or fentanyl (Hameln Pharma ApS, Glostrup, Denmark): the randomisation sequence was handled by the hospital pharmacy at Aarhus University Hospital using a centralised randomisation procedure (www.sealedenvelope.com). The hospital pharmacy prepared medical kits with identical appearance containing vials with the study drug (commercial, non-blinded methadone or fentanyl), isotonic saline for dilution of the study drug, a 10 ml syringe, a label for unique marking of the syringe with personal data and randomisation number, and a sealed envelope containing information of the actual study drug in case unblinding was clinically necessary. Two healthcare professionals (doctor or nurse) not involved in the trial or the treatment of the included participants prepared, marked, and controlled the study drug. Once prepared, the 10 ml colourless syringe containing either diluted methadone (concentration 2 mg ml^−1^) or fentanyl (concentration 30 μg ml^−1^) was given to one of the research team members and administered according to the protocol.

### Intraoperative management

All participants were anaesthetised according to a standardised regimen. Participants were given oral paracetamol (acetaminophen: 1000 mg) and NSAID (ibuprofen 400 mg) on the morning of surgery. Propofol and remifentanil were used for both anaesthetic induction (propofol 10 mg kg^−1^ h^−1^ and remifentanil 60 μg kg^−1^ h^−1^) and maintenance (propofol 5 mg kg^−1^ h^−1^ and remifentanil 30 μg kg^−1^ h^−1^) and titrated according to clinical judgement of anaesthetic depth. The tracheas of all participants were intubated orally and standard monitoring used (noninvasive blood pressure, peripheral oxygen saturation, and cardiac telemetry). Vasopressors, crystalloids, and neuromuscular blocking agents were administered at the discretion of the anaesthesiologist. The study drug was given as an i.v. dose (methadone: 0.2 mg kg^−1^ or fentanyl: 3 μg kg^−1^) according to ideal body weight (definition: height —105 cm for females and height—100 cm for males)). The administered dose reflects other clinical studies on methadone.^10^^,^^13^^,^^14^ The study drug was administered after induction of anaesthesia and before the start of surgery. In the present study, cold steel tonsillectomy with blunt metal instruments was utilised for peritonsillar space dissection, and haemorrhage control was typically achieved through compression, diathermy, and ligation. Preceding incision, local anaesthesia (lidocaine 10 mg ml^−1^ with epinephrine 5 μg ml^−1^) was infiltrated into the peritonsillar tissue (1 ml on each side) to mitigate perioperative bleeding and postoperative discomfort.

### Postoperative management

Pain was evaluated upon arrival at the postanaesthesia care unit (PACU) and roughly every 15 min using a numeric rating scale (NRS, 0–10; 0=no pain and 10=worst possible pain). The initial pain score at time 0 was recorded upon arrival at the PACU. PACU nurses were trained to use a standardised protocol for postoperative pain treatment. Moderate pain (NRS >3) was treated with an initial i.v. morphine dose of 0.1 mg kg^−1^, followed by titration of additional i.v. doses as needed. If further analgesia was required, clinicians could administer oral morphine (10 mg), additional i.v. morphine, or both, at their discretion until pain was adequately controlled (NRS ≤3). If the patient presented with a previous history of severe side-effects (e.g. pruritus, nausea or vomiting) or limited effect of morphine, oxycodone (both i.v. and oral) was given to the patient using a similar protocol and dosage to morphine. Additionally, fentanyl 50 ug i.v. was used if pain was severe (NRS >6). Taking the timing of the morning administration into consideration, oral paracetamol and NSAID (ibuprofen) was repeated during the PACU stay.

Postoperative nausea, vomiting, or both (PONV) prophylaxis and established PONV was treated according to the national Danish recommendations^15^: i.v. dexamethasone 8 mg and ondansetron 4 mg administered during surgery for participants with a low risk of PONV, and additional use of droperidol 0.25 mg in patients with a high risk of PONV (three or more risk factors for PONV: female sex, history of PONV or motion sickness, nonsmoker status, and expected postoperative opioid requirements).^16^ All participants were discharged from the PACU to a surgical short stay unit from where the majority of participants were discharged from hospital on the same day as surgery unless postoperative symptoms or complications occurred (postoperative bleeding, unacceptable pain, PONV etc.). After hospital discharge, pain treatment consisted of paracetamol (1000 mg four times daily), naproxen (500 mg twice daily), and oral morphine 10 mg as per needed (10 tablets prescribed for each patient). Participants were instructed to contact their general practitioner if they required pain treatment more than 14 days after surgery.

### Outcomes

The two joint primary outcomes were (1) pain (NRS, 0–10) at swallowing on PACU arrival and (2) the total postoperative opioid consumption from tracheal extubation to 5 days after surgery. Opioid consumption was reported and calculated as oral morphine equivalents.^17^ Secondary outcomes included (3) the time from arrival to discharge from PACU and hospital, (4) pain (NRS, 0–10) at swallowing 1, 2, 3, 5, and 7 days after surgery, (5) patient satisfaction (NRS, 0–10) and, (6) number of participants with a normal level of sedation (defined as awake and cooperative, oriented and tranquil on the Ramsay Sedation Scale^18^) 4 h after tracheal extubation, (7) PONV measured on a 4-point Likert scale (none/mild/moderate/severe) after 1, 2, and 3 days, (8) any adverse events during observation at the PACU (hypoventilation [ventilatory frequency <10 bpm] or hypoxaemia [peripheral oxygen saturation <90%]), and (9) the total postoperative opioid consumption from tracheal extubation to 24 h and 7 days. We recorded the following baseline data in order to describe the study population: age, sex, height, weight, ASA score, tobacco and alcohol consumption, and the duration of anaesthesia and surgery. While not a predefined outcome, pain at rest (NRS, 0–10) was also recorded at the same time points as pain at swallowing and is presented in the results for completeness.

### Sample size estimation and statistical analysis

The sample size was based on a pre-trial audit of 30 patients undergoing tonsillectomy in our department where the accumulated use of oral morphine equivalents after 5 postoperative days was 65 mg morphine. Based on the existing literature on perioperative methadone in various surgical populations,^9^^,^^10^^,^^13^^,^^14^^,^^19^^,^^20^ to detect a 40% reduction in administered oral morphine equivalents in the methadone group with 90% power (alpha 5%), ∼65 participants would be required in each group. Evaluating the pain reductions seen in other methadone studies,^9^^,^^10^ we arbitrarily considered this sample size to be sufficient to detect a clinically relevant difference of 2 units in postoperative pain intensity (NRS) throughout the study period.^21^ Study data were handled using an electronic data capture tool (REDCap).^22^

Data collection in the PACU was performed by nurses whereas all other post-discharge data were obtained by electronic questionnaires sent automatically by REDCap to every participant's e-mail. Participants were only contacted by telephone if questionnaires were not answered within 24 h. One reminder was sent 6 h after the first e-mail if the questionnaire was not completed. Data were exported to Stata software version 15.0 (StataCorp, College Station, TX, USA), with which statistical analyses were performed. To test the difference between groups over time, a mixed linear model fit for repeated measures was applied with pain and opioid consumption as dependent variables. Group and time and the interaction between them were included as fixed effects to assess both the main effects and their interaction over time. Patient identification was included as a random effect to account for the correlation between repeated measures within the same individual. The validity of the model was assessed by inspecting the distribution of residual plots. As *post hoc* analyses, standard tests were applied as described below. Pain intensity at rest was assessed after operation at the same time points as pain at swallowing (days 1, 2, 3, 5, and 7) to provide a more detailed description of postoperative pain. This analysis was conducted *post hoc* and is intended for descriptive purposes only. Medians with inter-quartile ranges (IQR) are presented for skewed data and compared with the Mann–Whitney test, whereas means with 95% confidence intervals were used for normally distributed data and compared with the Student's *t*-test. Categorical data were reported as numbers (%) and compared using the χ^2^ test. All *P*-values are two-sided and considered significant if <0.05.

## Results

**Fig 1:**
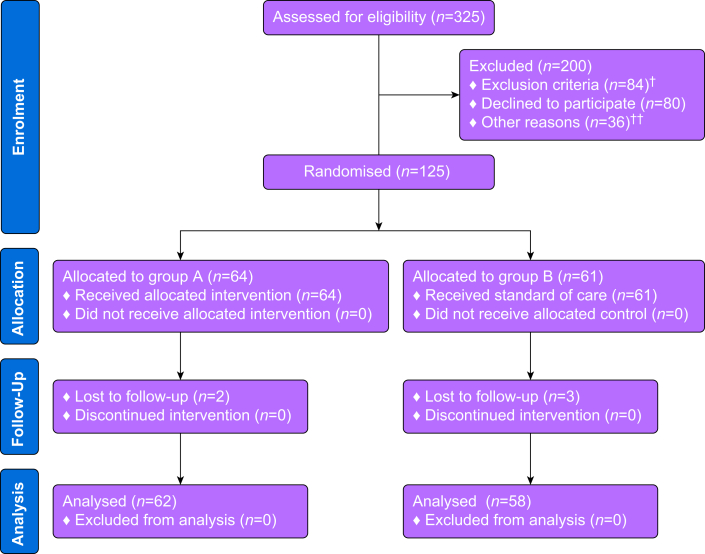
Participant flow diagram. In five instances, medical kits were opened, prepared, and labelled for administration as per protocol. However, as a result of unforeseen circumstances, such as cancellations or changes in participants' eligibility immediately before surgery, the prepared medication was not administered. As a result, these kits were considered wasted and could not be reused for other participants. ∗Patients were excluded because of preoperative treatment of any drug prolonging the QT interval (*n*=72), language problems (*n*=5), wasted medical kits (*n*=5), and preoperative opioid use (*n*=2). ^†^Other reasons included patients who were not seen in the pre-assessment clinic to receive study information (*n*=23) and patients who were seen in the pre-assessment clinic but did not receive information (*n*=13). Purple=Methadone, Blue=fentanyl.

The trial was conducted during a 12-month period from December 2022 to December 2023 with the flow of participants presented in Figure 1. Because of unforeseen circumstances in our investigator group we needed to terminate the trial before reaching the recruitment goals set out in the sample size calculation. A total of 125 participants were allocated to a group, of whom five were lost to follow-up, leaving 120 participants available for analysis with 62 participants treated with methadone and 58 participants treated with fentanyl. The groups were similar at baseline in terms of age, sex, ASA score, BMI, alcohol and tobacco use, and the length of surgery and anaesthesia (Table 1).

**Table 1:** Baseline characteristics of the patients. ASA, American Society of Anesthesiologists; BMI, body mass index; CI, confidence interval; IQR, inter-quartile range. ∗Tobacco use defined as daily use within last 6 weeks. ^†^Alcohol use defined as at least occasional use.

Group	Methadone	Fentanyl
*N*	62	58
Age (yr), median (IQR)	24 (20–30)	25.5 (22–30)
Female sex, *n* (%)	45 (72.6)	41 (70.7)
ASA score, *n* (%)		
1	44 (71.0)	39 (67.2)
2	16 (25.8)	19 (32.8)
3	2 (3.2)	0 (0)
BMI (95% CI)	25.0 (23.7–26.3)	26.2 (24.6–26.6)
Tobacco use∗, *n* (%)	7 (11.3)	9 (15.5)
Alcohol use^†^, *n* (%)	37 (59.7)	33 (56.9)
Duration of surgery (min) (IQR)	35 (24–49)	35 (28–49)
Duration of anaesthesia (min) (IQR)	71.5 (58–85)	70 (61.5–80)
Dosage (mg μg^−1^), median (IQR)	13.8 (12.4–15.6)	210 (195–225)

**Fig 2:**
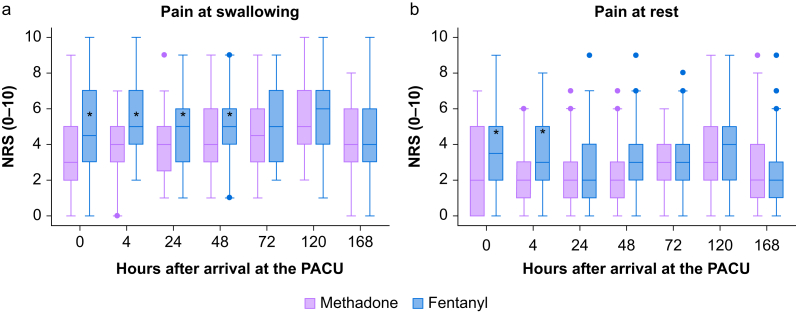
(a) Pain intensity at swallowing. Timeline defined as hours after arrival at the PACU. Boxes represent median values with lower and upper quartile ranges. Whiskers represent minimum and maximum data values and outliers are given as dots. Pain scores were lower in the methadone group immediately after extubation (*P*=0.02), after 4 h (*P*=0.0004), 24 h (*P*=0.005), and 48 h (*P*=0.03). (b) Pain intensity at rest. Timeline defined as hours after arrival at the PACU. Boxes represent median values with lower and upper quartile ranges. Whiskers represent minimum and maximum data values and outliers are given as dots. Pain scores were lower in the methadone group upon arrival at the PACU (*P=*0.01) and after 4 h (*P*=0.0009). NRS, numeric rating scale; PACU, postanaesthesia care unit. ∗Statistically significant difference (*P*<0.05). Purple=Methadone, Blue=fentanyl.

Postoperative pain intensity at swallowing is presented in Figure 2a. There was overall evidence against the hypothesis of parallel curves (*P*-value 0.0005). Regarding the first primary outcome, pain intensity at swallowing was significantly lower in the methadone group compared with the fentanyl group upon arrival at the PACU (3 [IQR 2–5] *vs* 4.5 [IQR 3–7], *P*-value 0.02) and remained lower 4 h (4 [IQR 3–5] *vs* 5 [IQR 4–7], *P*=0.0004), 24 h (4 [IQR 2.5–5] *vs* 5 [IQR 3–6], *P*=0.005) and 48 h (4 [IQR 3–6] *vs* 5 [IQR 4–6], *P*=0.03) after surgery. However, the observed differences were smaller than the predefined clinically relevant difference of at least 2 units on NRS. Similar but less pronounced differences were observed for pain at rest (Fig. 2b).

**Fig 3:**
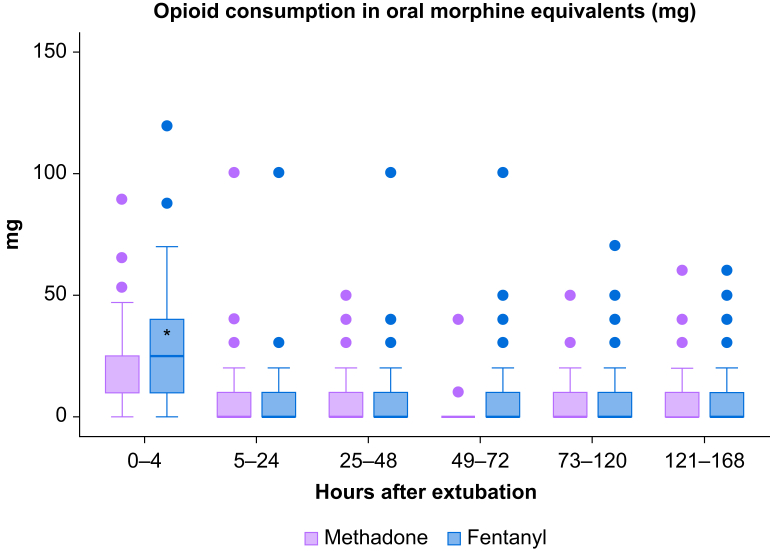
Postoperative opioid consumption in oral morphine equivalents. Timeline defined as hours after extubation. Boxes represent median values with lower and upper quartile ranges. Whiskers represent minimum and maximum data values and outliers are given as dots. Opioid consumption was lower in the methadone group 4 h after extubation (*P*=0.003). ∗Statistically significant difference (*P*<0.05). Purple=Methadone, Blue=fentanyl.

Postoperative opioid consumption is presented in Figure 3. There was a significant difference between the two groups over time (*P*-value 0.02). Participants in the methadone group had significantly lower cumulative total opioid consumption in oral morphine equivalents for the first 5 postoperative days, ∼40% less than those treated with fentanyl (30 mg [IQR 10–50 mg] *vs* 49 mg [IQR 29–80 mg], *P*-value 0.002). However, this difference was predominantly observed in the PACU (10 mg [IQR 10–25 mg] *vs* 25 mg [IQR 10–40 mg], *P*-value 0.003), with similar consumption in both groups in subsequent days. There were no differences in patient satisfaction, length of hospital stay, sedation, and the number of other adverse events in the PACU, whereas significantly more methadone participants suffered from moderate to severe PONV at 24 h (45.0% *vs* 14.1%, *P*=0.001), at 48 h (43.3% *vs* 17.6%, *P*=0.005) and after 72 h (33.9% *vs* 18.2%, *P*=0.03) (Table 2). Among the study population, 40 participants did not require opioids beyond the PACU, with no statistically significant difference observed between the methadone group (24 participants, 38.7%) and the fentanyl group (16 participants, 27.6%; *P*=0.20).

**Table 2:** Postoperative patient satisfaction, complications, and length of stay. NRS, numeric rating scale; PACU, postanaesthesia care unit; PONV, postoperative nausea and vomiting. Definitions: patient satisfaction is reported on a numeric rating scale (NRS) with inter-quartile ranges (IQR). ∗PONV defined as either moderate or severe on a 4-point Likert scale (none, mild, moderate, severe). ^†^Normal level of consciousness defined on the Ramsay Sedation Scale as awake and cooperative, oriented, and tranquil. ^‡^Hypoventilation defined as a ventilatory frequency <10 bpm. ^¶^Hypoxemia defined as an oxygen saturation <90%. ^§^The accumulated time spent in the PACU and a surgical short stay unit.

Group	Methadone	Fentanyl	*P-*value
	*n*=62	*n*=58	
Patient satisfaction (NRS, 0–10)			
Day 1	9 (8–10)	9 (8–10)	0.44
Day 7	8 (6–9)	8 (5–9)	0.62
∗PONV day 1, *n* (%)			
None	20 (33.3)	37 (64.9)	0.001
Mild	13 (21.7)	12 (21.0)	
Moderate	14 (23.3)	5 (8.8)	
Severe	13 (21.7)	3 (5.3)	
∗PONV day 2, *n* (%)			
None	15 (25.0)	28 (49.1)	0.005
Mild	19 (31.7)	19 (33.3)	
Moderate	14 (23.3)	8 (14.1)	
Severe	12 (20.0)	2 (3.5)	
∗PONV day 3, *n* (%)			
None	17 (27.4)	25 (45.5)	0.03
Mild	24 (38.7)	20 (36.6)	
Moderate	15 (24.2)	10 (18.2)	
Severe	6 (9.7)	0 (0.0)	
Normal level of consciousness^†^ in PACU, *n* (%)	47 (75.8)	47 (82.5)	0.37
Adverse events in PACU, *n* (%)			
Hypoventilation^‡^	2 (3.2)	3 (5.3)	0.46
Hypoxemia^¶^	2 (3.2)	2 (3.5)	0.66
Length of stay (h) (IQR)			
PACU	2.1 (1.7–2.4)	2.1 (1.8–2.5)	0.47
Hospital^§^	5.7 (3.8–6.9)	4.7 (3.8–5.7)	0.20
Discharge same day as surgery, *n* (%)	48 (80.0)	49 (89.1)	0.18

## Discussion

In this randomised controlled trial on adult patients undergoing elective bilateral tonsillectomy, a single dose of intraoperative methadone reduced cumulative total postoperative opioid consumption by ∼40% over the first 5 days after surgery. This reduction was primarily because of lower opioid use in the first 24 h, with similar consumption between groups in the remaining observation period. Pain intensity at swallowing was less in the methadone group for the first 2 days compared with fentanyl, but did not reach a clinically relevant difference of 2 units on the NRS scale. No differences in terms of patient satisfaction, adverse events in the PACU, and length of hospital stay were observed, but despite the reduced consumption of opioids in the postoperative phase, more patients treated with methadone reported moderate or even severe PONV in the first 3 postoperative days.

A recent small paediatric study on elective tonsillectomies found similar reductions in postoperative opioid requirements compared with fentanyl but failed to find any difference in postoperative pain or side-effects.^23^ Although the surgical procedure was the same as in our study, numerous aspects of pain such as perception, treatment approach, and outcome reporting are different between children and adults, which make comparison of results difficult. Our results are in line with a systematic review including data from 13 studies investigating the role of i.v. methadone in the treatment of acute pain across various surgical specialties.^8^ Postoperative opioid requirements were reported to be significantly lower in the group of patients treated with methadone for as long as 72 h after surgery but with huge variation in the actual differences (i.v. morphine equivalents from 0.5 mg to 53 mg). In addition, significantly lower pain scores during activity were reported for methadone in the first 72 postoperative hours, and with similar but less pronounced differences for pain scores at rest.

The effect of methadone might be associated with the extent of surgery. In a randomised study on patients scheduled for major heart surgery, 156 patients received perioperative methadone or fentanyl.^9^ The study found that opioid requirements could be 40% lower in the first 24 postoperative hours with methadone and, even more noteworthy, that patients were more satisfied and had less pain for the first 72 h after surgery without increased rates of opioid-related complications such as nausea, vomiting, sedation, itching, and ventilatory deterioration.

The same group of researchers randomly allocated 120 patients undergoing major spine surgery to intraoperative methadone or hydromorphone, finding postoperative opioid consumption reduced by >50% in the first 3 days after surgery.^10^ Additionally, pain intensity was significantly less in the methadone group in essentially all observations in the first 72 h after surgery. There was no difference in terms of side-effects and all these findings probably contributed to a higher degree of satisfaction among patients treated with methadone.

While surgical populations exposed to extensive surgical stimuli seem to gain from methadone single-shot therapy, the benefits in smaller operations, such as laparoscopic surgery, seem less clear with no or clinically irrelevant reductions in postoperative opioid requirements^24^^,^^25^ and pain intensity.^20^^,^^24^, ^25^, ^26^ Even though advantages of perioperative methadone have been observed in small studies on cardiac^27^ and spine surgery,^13^ bariatric surgery,^28^ and abdominal surgery,^29^ numerous methodological issues question the true effect of the intervention.

Contrary to the superior effect on pain intensity and decreased usage of postoperative opioid consumption documented in our study, more patients in the methadone group suffered from moderate to severe PONV for 3 days after surgery. This observation highlights an important trade-off between methadone's potential benefits in reducing pain and opioid consumption and its notable drawback of an increased PONV incidence. For patients undergoing tonsillectomy—a procedure often associated with significant postoperative discomfort—this trade-off may carry different weight depending on individual tolerance to nausea and vomiting, and personal pain management priorities. The prevalence of PONV in methadone studies varies widely (5.5–80%) but is typically reported at 30–50%.^9^^,^^10^^,^^19^^,^^26^^,^^27^ This aligns with our findings for the methadone group (∼40–45%) but not for the fentanyl group (∼14–18%) or the broader surgical population, where ∼28% experience PONV after operation.^30^ One key question arising from this study is whether the observed reduction in pain intensity and postoperative opioid consumption with methadone justifies the higher incidence of PONV reported by nearly half of the patients in the methadone group. While reducing pain and opioid use are significant objectives, patients' perceptions and tolerance of PONV vary substantially.^31^ Regardless, comprehensive efforts should always be made to alleviate PONV for surgical patients as nausea and vomiting appear to be some of the most unwanted postoperative side-effects.^32^

This study has several limitations that need to be addressed and taken into account upon interpretation. First, a considerable number of patients were excluded at the screening stage (Fig. 1) with an associated risk of selection bias. Second, the preparing of the study drug on site may introduce errors and accidental unblinding compared with hospital pharmacy drug preparation before clinical use. Third, electronic questionnaire-based follow-ups can introduce misinterpretations of questions, missing data or recall bias if not completed at the exact postoperative time points. A pragmatic approach was finalised during the study design phase to collect data at specific postoperative timepoints to analyse the outcomes of interest. This was chosen to minimise the inconvenience and burden to study participants during the follow-up period. Consequently, data were collected on non-consecutive postoperative days which may have introduced recall bias for missing days and decreased the cohesiveness of data collected. Finally, one may argue that the comparison of methadone with a short-acting drug such as fentanyl was not equipotent and that the impact on postoperative pain and opioid consumption was predictable. However, the current study also investigated potential, undesirable side-effects (e.g. PONV) and the comparison reflected a normal everyday approach to same-day surgical patients. The reported conversion factors for methadone in the literature vary greatly and are mainly based on small retrospective studies on chronic pain patients or patients with substance use disorders receiving opioid agonist therapy.^33^ In those patient cohorts, great variability in the hepatic clearance of methadone, increased risk of accumulation with repeated dosing, and development of opioid tolerance adds substantial uncertainty to dose conversions between methadone and other opioids. However, these factors are not present in our study cohort of healthy, opioid-naive participants who were given a single dose of i.v. methadone.^34^ Considering these differences, the chosen doses of methadone (0.2 mg kg^−1^) and fentanyl (3 μg kg^−1^) appear equipotent based on prior similar studies^10^^,^^13^^,^^14^ and WHO guidelines on single-dose methadone to morphine equivalence.^35^

Additionally, a recent dose-finding pilot study on next-day discharge outpatient surgery suggested that doses above 0.1 mg kg^−1^ of i.v. methadone are necessary to effectively manage acute postoperative pain, while 0.25 mg kg^−1^ ideal body weight may be the optimal dose to reduce opioid consumption and control pain without causing unwanted side-effects.^36^ Despite concerns about respiratory depression with methadone, our findings revealed no significant difference in PACU adverse events between methadone and fentanyl. This, combined with the relatively short anaesthesia duration of ∼1 h, underscores methadone's relative safety for use in adult tonsillectomy patients.

In conclusion, one dose of intraoperative methadone decreased pain intensity for the following 2 days after surgery and postoperative opioid consumption was reduced by almost 40% for the first 5 postoperative days. However, a significant proportion of patients suffered from moderate to severe PONV for 3 days after surgery. Based on these results, we do not advocate for the use of methadone for all tonsillectomy patients in general, but careful patient selection and individualised preoperative discussions could identify subgroups who might benefit most from methadone's analgesic properties despite its associated risks. Future investigations of methadone should aim to identify and treat patients at particular risk of post-surgical pain, as the benefits of reducing pain in these patients might overshadow the drawbacks of experiencing PONV.

## Authors’ contributions

Included the patients, undertook GCP monitoring on site: MB

Supported the daily conduct of the trial, and drafted, revised, and approved the final manuscript: MB, PU

Responsible for the final GCP monitoring: PU

Responsible for data entering to RedCap, contributed to everyday trial conduction: TKa

Revised and approved the final manuscript: TKa, MR, TKj, RTS, TEK, LN

Contributed to the design of the trial and supported the conduction: MR

Took part in the initial trial design and selection of surgical population, drafted parts of the manuscript: TKj

Supported the trial design, facilitated trial conduction: RTS

Contributed to the study design: TEK

Designed and supervised the study, contributed with data interpretation: LN

Designed the study, obtained all approvals, build the electronic capture tools, undertook the statistical analyses and drafted the manuscript: KDF

## Funding

A project grant from Health Research Foundation of Central Denmark Region (A3639) and from the Danish Society of Anesthesiology & Intensive Care Medicine.

## Declarations of interest

The authors declare that they have no conflicts of interest.
